# Clinical characteristics of patients newly diagnosed with COPD by the fixed ratio and lower limit of normal criteria: a cross-sectional analysis of the TargetCOPD trial

**DOI:** 10.2147/COPD.S146914

**Published:** 2018-06-21

**Authors:** Martin R Miller, Shamil Haroon, Rachel E Jordan, Alice J Sitch, Andrew P Dickens, Alexandra Enocson, David A Fitzmaurice, Peymané Adab

**Affiliations:** 1 Institute of Applied Health Research, College of Medical and Dental Sciences, University of Birmingham, Edgbaston, Birmingham, UK; 2 Warwick Clinical Trials Unit, Warwick Medical School, University of Warwick, Coventry, UK

**Keywords:** lower limit of normal, diagnostic criteria, primary care

## Abstract

**Background:**

Consensus on the definition of airflow obstruction to diagnose COPD remains unresolved.

**Methods:**

We undertook systematic case finding for COPD in primary care using the fixed ratio (FR) criterion (forced expiratory volume in 1 s/forced vital capacity [FEV_1_/FVC] <0.7) for defining airflow obstruction and also using the lower limit of normal (LLN). We then compared the clinical characteristics of those identified by the 2 criteria.

**Results:**

A total of 3,721 individuals reporting respiratory symptoms were invited for spirometry. A total of 2,607 attended (mean age 60.4 years, 52.8% male, 29.8% current smokers) and 32.6% had airflow obstruction by FR (“FR+”) and 20.2% by LLN (“LLN+”). Compared with the LLN+/FR+ group, the LLN−/FR+ group (12.4%) was significantly older, had higher FEV_1_ and FEV_1_/FVC, lower COPD assessment test scores, and less cough, sputum, and wheeze, but was significantly more likely to report a diagnosis of heart disease (14.2% versus 6.9%, *p*<0.001). Compared with the LLN+/FR+ group, the LLN−/FR− group was younger, had a higher body mass index, fewer pack-years, a lower prevalence of respiratory symptoms except for dyspnea, and lower FVC and higher FEV_1_. The probability of known heart disease was significantly lower in the LLN+/FR+ group compared with those with preserved lung function (LLN−/FR−) (adjusted odds ratio 0.62, 95% CI: 0.43–0.90) but this was not seen in the LLN−/FR+ group (adjusted odds ratio 0.90, 95% CI: 0.63–1.29).

**Conclusion:**

In symptomatic individuals, defining airflow obstruction by FR instead of LLN identifies a significant number of individuals who have less respiratory and more cardiac clinical characteristics.

## Introduction

COPD is the third leading cause of premature mortality and the fifth leading cause of disability adjusted life years globally.^1^ Huge efforts have been made to improve the diagnosis of COPD in primary care. However, the definition of COPD remains an unresolved issue with controversy remaining about the criteria for defining airflow obstruction. The Global Initiative for Obstructive Lung Disease (GOLD) and the National Institute for Health and Care Excellence (NICE) in the UK recommend the use of a fixed ratio (FR) of forced expiratory volume in 1 s (FEV_1_) to forced vital capacity (FVC) of <0.7 as the diagnostic threshold for airflow obstruction.^2^,^3^ However, this criterion does not take into consideration that FEV_1_/FVC declines with age and differs between the sexes and by ethnicity.^4^

A number of epidemiological analyses suggest that using the FR criterion misclassifies a significant proportion of healthy older men as having airflow obstruction and under-diagnoses younger females.^5^ There is also particular concern that using this criterion misclassifies patients with breathlessness due to cardiovascular disease^6^ who then may miss out on necessary treatment, while receiving inappropriate medication and potentially adverse outcomes.^7^,^8^ The European Respiratory Society (ERS) and American Thoracic Society (ATS) recommend using a more statistics-based definition of airflow obstruction derived from population-based reference values.^9^ This defines individuals with an FEV_1_/FVC below the lower limit of normal (LLN; below the fifth percentile adjusted for age, sex, height, and ethnic group), as having airflow obstruction.^10^ There is increasing evidence that this approach correlates better with clinical outcomes than the FR.^6^

With increasing emphasis on actively case finding undiagnosed COPD,^2^ there is potential for large numbers of new cases to be diagnosed.^11^ In this context, it is important that misdiagnosis is avoided and those more likely to benefit are identified. A large randomized controlled trial was conducted in the West Midlands, UK, evaluating the effectiveness and cost-effectiveness of COPD case finding in primary care.^12^,^13^ This trial offered spirometry to subjects with respiratory symptoms aged >40 years. The objective of the present study was to compare the clinical characteristics of symptomatic patients in primary care with case-found COPD diagnosed when using the FR criterion with those identified when using the LLN.

## Methods

### Study design

This is a post hoc cross-sectional analysis of data from TargetCOPD, which was a cluster-randomized controlled trial based in primary care that compared 2 approaches to COPD case finding against usual care. Full details of the trial have been previously described.^12^ In the case-finding arm, patients aged 40–79 years with no prior diagnosis of COPD were eligible. Participants were provided with respiratory questionnaires that ascertained information on demographic characteristics, symptoms, smoking history, and self-reported comorbidities. Responders reporting respiratory symptoms (either chronic cough/phlegm for ≥3 months for at least 2 years, or wheeze in the last 12 months, or Medical Research Council [MRC] grade 2 dyspnea or worse) were invited for a spirometry assessment. The current analysis used data from subjects recruited in practices randomized to the case-finding arm of the trial who attended a spirometry assessment.

### Setting

The case-finding arm of TargetCOPD included 27 general practices from the West Midlands, UK, with participation from August 2012 to June 2014.

### Participants

All subjects included in this analysis had participated in the case-finding arm of TargetCOPD, had reported at least 1 respiratory symptom (as detailed previously), had provided written informed consent, and had attended a spirometry assessment. Subjects were identified through electronic searches of general practice registers. Initially, only ever smokers were eligible, although due to difficulties with accurate identification from primary care health records, never smokers were also included. General practitioners could exclude patients at their discretion if, for example, they had dementia, a recent bereavement, or terminal diagnosis.

### Spirometry assessment

Spirometry was performed by research assistants who had been trained using a short modified program modeled on the Association of Respiratory Technologists and Physiologists spirometry course at the lung function laboratory at Queen Elizabeth Hospital Birmingham. The research assistants also received additional training every 6 months during the course of the study. Post-bronchodilator spirometry was performed according to ATS and ERS guidelines using EasyOne ultrasonic flow head spirometers (ndd Medical Technologies, Zurich) with bespoke software (developed by MRM). Four 100 µg doses of salbutamol were administered via a Volumatic spacer 20 min prior to performing spirometry. Every spirometry trace was over-read and quality assured by a lung function specialist (MRM) and spirometers underwent daily calibration checks. Patients’ height was measured to the nearest centimeter using a portable stadiometer (or estimated from arm-span where necessary).

### Diagnosis of COPD

COPD was defined as having airflow obstruction according to 2 separate criteria, among those with respiratory symptoms (as described previously):

1. Post-bronchodilator FEV_1_/FVC<LLN, defined as a *z*-score for FEV_1_/FVC below the fifth percentile from the GLI 2012 lung function reference equations (“LLN criterion”).^10^
2. Post-bronchodilator FEV_1_/FVC<0.7 (“FR criterion”).

### Data collected

Data from self-reported questionnaires were available on sociodemographic characteristics (age, sex, ethnic group, and socioeconomic status), smoking status (including pack-years), self-reported comorbidities (hypertension, heart disease, heart failure, diabetes, stroke, lung cancer, tuberculosis, and depression), respiratory symptoms (cough, phlegm, wheeze, dyspnea, and rhinorrhea), COPD assessment test (CAT) scores, and overall quality of life (EQ-5D).

### Statistical methods

The sociodemographic and clinical characteristics of subjects were summarized separately according to the following 4 diagnostic groupings:

1. Normal spirometry according to both LLN and FR criteria (LLN−/FR−).
2. Airflow obstruction according to both criteria (LLN+/FR+).
3. Airflow obstruction according to the FR but not LLN (LLN−/FR+).
4. Airflow obstruction according to LLN but not the FR (LLN+/FR−).

Continuous variables were summarized as means and SDs and categorical variables as percentages. The FEV_1_, FVC, and FEV_1_/FVC ratio were summarized as *z*-scores using GLI 2012 equations.^10^ Clinical characteristics were compared across groups using Kruskal–Wallis H-tests for continuous variables (as they had skewed distributions), and chi-squared tests for categorical variables. The main comparisons were bivariate analyses between the LLN+/FR+ and LLN−/FR+ groups, and between the LLN+/FR+ and LLN−/FR− groups, comparing the prevalence of each characteristic. The *p*-value thresholds for statistical significance were adjusted for multiple testing using the Bonferroni correction.^14^

Since previous literature has suggested that use of the FR tends to include more patients with cardiovascular disease than the LLN criteria,^6^ we tested this hypothesis in our data using a logistic regression model with self-reported cardiovascular disease (composite outcome of self-reported heart disease and/or heart failure) as the outcome, and LLN/FR status as an independent variable with models derived adjusting for age, sex, smoking status, self-reported diabetes mellitus, and hypertension.

### Ethical approval

Research governance and ethics approval for TargetCOPD was provided by the Solihull Research Ethics Committee (Ref: 11/WM/0403).

## Results

**Figure 1:**
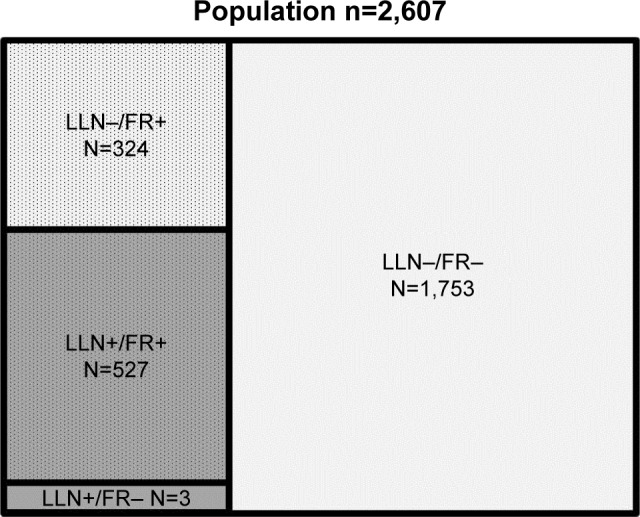
Number of participants in the 4 diagnostic groups. **Note:** For clarity, the dimensions are not exactly to scale. **Abbreviations:** FR, fixed ratio (FEV_1_/FVC<0.7); LLN, lower limit of normal (FEV_1_/FVC<5th percentile); FEV_1_, forced expiratory volume in 1 s; FVC, forced vital capacity.

The case-finding arm of TargetCOPD had 32,811 eligible participants. Questionnaires were given to 22,116 and 7,778 responded, of whom, 4,355 (56%) reported respiratory symptoms. From those with symptoms, 3,721 were invited for a spirometry assessment and 2,607 (70%) attended and were included in the current analysis (Figure 1).

The mean age of the included 2,607 participants was 60.4 years, 52.8% were male, and 29.8% were current smokers. The demographic characteristics, smoking status, BMI, and spirometry results are summarized in Table 1, stratified by diagnostic criteria for airflow obstruction. There were 851 (32.6%) individuals with airflow obstruction according to the FR, of whom, 527 (20.2%) met the LLN criterion (LLN+/FR+). A total of 324 (12.4%) had airflow obstruction only by the FR but not by the LLN (LLN−/FR+), and 1,753 (67.2%) did not meet the criteria for airflow obstruction by either criteria (Figure 1). Not included in Table 1 are 3 individuals with airflow obstruction by the LLN but not identified by the FR criterion. They were all younger females (mean age 46.1 years), with a mean pack-year history of 2.9, BMI of 31.3, and FEV_1_/FVC *z*-score of −1.73.

Compared with the LLN+/FR+ group, patients in the LLN−/FR+ group were older and had better lung function (higher FEV_1_ and FEV_1_/FVC *z*-scores). A higher proportion was male, although this difference was not statistically significant. However, smoking pack-years was similar between the LLN+/FR+ and LLN−/FR+ groups, both of which were significantly higher than for subjects with preserved lung function (LLN−/FR−). Compared with the LLN+/FR+ group, subjects in the LLN−/FR− group were younger, and had a higher BMI, fewer pack-years, and worse zFVC but higher zFEV_1_ and zFEV_1_/zFVC.

Table 2 summarizes the symptoms, self-reported comorbidities, and quality of life for participants, stratified by diagnostic group. Symptoms of cough, sputum, and wheeze were all more prevalent in the LLN+/FR+ group compared with the LLN−/FR+ and the LLN−/FR− groups. CAT scores were 2 points higher (equivalent to the minimum clinically significant difference^15^) in the LLN+/FR+ than the LLN−/FR+ group, although there was a substantial amount of missing data (12.8%) for this variable. However, there were no significant differences in overall quality of life (as measured by EQ-5D). In contrast to the LLN+/FR+ group, the overall symptom burden in the LLN−/FR+ group was similar to the unobstructed group (LLN−/FR−), except for a slightly higher prevalence of wheeze. The 3 patients with airflow obstruction by the LLN but not by the FR criterion were significantly more symptomatic than the other groups with a mean CAT score of 17.

**Figure 2:**
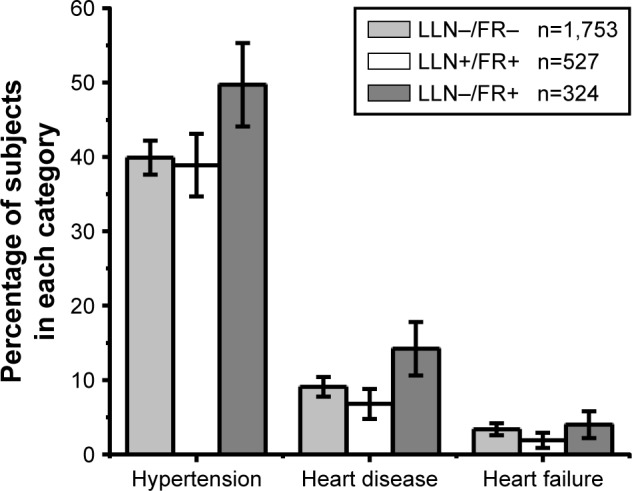
Prevalence of cardiovascular disease by diagnostic group. **Abbreviations:** FR, fixed ratio (FEV_1_/FVC<0.7); LLN, lower limit of normal (FEV_1_/FVC<5th percentile); FEV_1_, forced expiratory volume in 1 s; FVC, forced vital capacity.

The prevalence of heart disease was significantly higher in the LLN−/FR+ group compared with the LLN+/FR+ group (14.2% versus 6.9%, *p*<0.001) but the LLN−/FR+ group also had a significantly lower prevalence of depression (Table 2; Figure 2). The prevalence of asthma was significantly higher in the LLN+/FR+ group compared with the LLN−/FR− group (30.9% versus 18.4%, *p*<0.001). While this was also observed when comparing the LLN+/FR+ with the LLN−/FR+ group, this difference did not reach statistical significance. After adjusting for age and sex, the LLN+/FR+ group had a significantly lower probability of having heart disease compared with the LLN−/FR− group (odds ratio 0.57, 95% CI: 0.40–0.81) but no significant difference was found for the LLN−/FR+ group (Table 3). The same associations were found after adjusting for smoking status, self-reported diabetes mellitus, and hypertension.

## Discussion

### Main findings

Within a primary care population of previously undiagnosed adults with chronic respiratory symptoms, using the FR to define airflow obstruction resulted in a higher proportion being classified as having COPD, compared with using the LLN criterion (32.6% versus 20.2%, respectively). Those diagnosed with COPD by the FR but not by LLN were older, and had better lung function, lower symptom burden, a higher prevalence of self-reported cardiovascular disease, and a lower prevalence of depression, compared with those classified as having COPD by both criteria. Those diagnosed with COPD by LLN were also significantly more likely to have a past history of asthma than those without airflow obstruction. In addition, using the LLN classified a small number of younger, very symptomatic females as having COPD that the FR excluded. Finally, symptomatic individuals with preserved lung function appeared to have a higher probability of having heart disease and a lower FVC than those with airflow limitation by LLN.

### Relationship to other studies

The potential for over-diagnosis of COPD using the FR has been previously demonstrated. A population prevalence study of COPD in England and Wales for ages 40–95 years found FR-defined airflow obstruction in 22% of subjects but in only 13% when using the LLN criterion.^16^ Our findings also agreed with an analysis of the CanCOLD study, a large prospective population-based cohort study of COPD in Canada.^6^ This concluded that use of the FR for diagnosing airflow obstruction could lead to the misdiagnosis of older males with a history of cardiovascular disease. The authors proposed using an FEV_1_<80% predicted as an additional criterion for restricting the diagnosis to patients with a clinically more significant degree of airflow obstruction. Indeed, this approach was previously recommended by NICE.^3^ However, this approach still does not adequately account for variation in lung function by age, sex, and height, and therefore, can still lead to misclassification.^17^ Using FEV_1_<LLN as an alternative criterion would avoid this bias.

The Austria Burden of Obstructive Lung Disease study similarly evaluated whether the diagnostic criterion for airflow obstruction was associated with the prevalence of heart disease.^18^ Among 1,258 screened adults aged >40 years (with and without symptoms), 27% of LLN−/FR+ cases had self-reported heart disease compared to 15% of LLN+ cases.

It has been suggested that the LLN−/FR+ cases have COPD with heart disease as a comorbidity, which may account for their poorer prognosis in terms of higher risk of hospitalization and premature mortality.^19^ However, the observation that these individuals are older with a higher comorbidity burden, and yet have better lung function,^20^ suggests that heart disease may be their primary diagnosis. COPD is characterized by gradual and inexorable decline in lung function with increasing dyspnea and those with relatively good lung function would not be expected to have a high rate of early mortality from COPD.

It is well recognized that the FR criterion will not only over-diagnose airflow obstruction in the older population but also miss the diagnosis in younger females.^5^,^6^,^21^,^22^ In the current study, the 3 highly symptomatic females who were misclassified as having no airflow obstruction using the FR potentially have a lot to gain from clinical intervention.

A recent study of patients with COPD using a FR definition of airflow obstruction^23^ found marked differences in how subjects were classified by the 2011 GOLD symptom-related classification (A, B, C, and D)^24^ depending on whether the modified MRC (mMRC) or CAT scores were used to assess subjects. Use of the CAT score tended to reduce the prevalence of cardiac comorbidities in the 2 more severe groupings. The mMRC rating of dyspnea is not specific to respiratory disease whereas the CAT score measures disease-specific quality of life and includes 7 other domains, some being more specific to COPD. This lends weight to the possibility that a combination of mMRC dyspnea and FEV_1_/FVC<0.7 may over-represent primary cardiac disease rather than COPD. Related to this, our study found that the prevalence of dyspnea was similar between individuals diagnosed with COPD by either diagnostic criterion whereas cough and sputum were less prevalent in the LLN−/FR+ group.

Guder et al prospectively studied 405 individuals aged >65 years with a GP diagnosis of COPD.^25^ They examined FR and LLN criteria for airflow obstruction and compared this with a COPD diagnosis by an expert panel that had access to clinical information, radiology, and lung function. Both criteria misclassified patients when using the expert panel diagnosis as the reference standard. The FR criterion was associated with more false positive diagnoses and the LLN criterion with more false negatives. Expert panel diagnosis was a better predictor of exacerbations, hospitalizations, and mortality, than either the FR or LLN criteria, highlighting the importance of a holistic assessment in addition to lung function measurement for making an accurate diagnosis and predicting prognosis.

### Strengths and limitations

Our study had a large sample of symptomatic subjects recruited from primary care who are likely to be representative of those who would be targeted for COPD case finding. The study employed post-bronchodilator spirometry with rigorous quality assurance and collected a broad range of data on symptoms, comorbidities, and health-related quality of life, and accounted for multiple statistical comparisons.

Comorbidities were self-reported and not based on health records or objective measures. We also did not account for potential undiagnosed comorbidities. Current and former smokers appeared to have a lower risk of self-reported cardiovascular disease than never smokers, although this association was not statistically significant. The reason for this is unclear but may be because cardiovascular disease was self-reported, which would not account for undiagnosed cardiovascular disease.

There was also a significant amount of missing data for the CAT (12.8% missing) and MRC dyspnea (4.1% missing) scores, and no data on objective measures of exercise tolerance, lung imaging, gas transfer, or cardiac function were collected. Finally, only 35% of all eligible participants responded to the respiratory questionnaire and the findings are therefore potentially prone to responder bias.

### Implications for practice, policy, and research

Use of the FR for defining airflow obstruction may lead to the inclusion of a significant number of older patients with a history of cardiovascular disease as having COPD. This could lead to symptoms related to cardiovascular disease being falsely attributed to airflow obstruction, resulting in an inappropriate management strategy. It is particularly important that symptomatic patients with an FEV_1_/FVC above the LLN (irrespective of whether the FEV_1_/FVC is <0.7) are diagnostically assessed for cardiovascular causes of their symptoms and exacerbations. Cardiovascular morbidity and mortality are higher in people with COPD than they are in the general population and cardiovascular disease is a more common cause of death among these individuals than COPD itself.^26^ Furthermore, the early mortality in those with an FEV_1_/FVC<0.7 and with mild reduction in FEV_1_ has been found to be unexpectedly high due to cardiac disease.^20^ It will be important to investigate whether cardiovascular disease is predominantly responsible for the symptom burden experienced by these patients. This could include more detailed investigations for cardiovascular disease, including echocardiography.

Further research is also needed to understand the clinical implications and long-term outcomes of pursuing standard treatment for COPD in the LLN−/FR+ group. The burden of respiratory symptoms was generally lower in this group compared with those with airflow obstruction by LLN. This may potentially explain the difference in prevalence of depression, since these physical symptoms may contribute to poor mental health. However, this requires further investigation. Further investigation is also needed to understand the significantly high burden of self-reported asthma seen in those with airflow limitation by LLN.

The growing body of evidence that using the FR has the potential to over-diagnose COPD in older people with cardiovascular disease and underdiagnose younger females should be more fully acknowledged in clinical guidelines, with a recommendation to use the LLN criterion to help differentiate patients with COPD from those with a potential cardiovascular cause for breathlessness. This will be particularly important in the context of systematic case finding, where there is a significant risk of misdiagnosing large numbers of people.

## Conclusion

Use of the FR for defining airflow obstruction may lead to the inclusion of a significant number of older people with breathlessness as having COPD, who may, in fact, have age-related changes in lung function in the presence of cardiovascular disease as the cause for their symptoms. Further research is needed to assess the long-term outcomes and clinical implications of using the FR versus the LLN for diagnosing COPD and characterize cardiovascular health in symptomatic subjects with lung function that is within the normal population reference range.

## Figures and Tables

**Table 1:** Demographic characteristics and spirometry results stratified by diagnostic criteria

Participant characteristics	LLN+/FR+		LLN−/FR+		LLN−/FR−
	N (%)^a^	*p*-value^ψ^	N (%)^a^	*p*-value^$^	N (%)^a^
Subjects	527		324		1,753
Male	292 (55.4)	0.056	196 (60.5)	0.145	889 (50.7)
Age (years)					
Mean (SD)	61.5 (9.8)	0.0001*	68.6 (7.2)	0.0001*	58.6 (10.5)
40–49	81 (15.4)		3 (0.9)		436 (24.9)
50–59	139 (26.4)		41 (12.7)		523 (29.8)
60–69	185 (35.1)		130 (40.1)		484 (27.6)
70–79	122 (23.1)		150 (46.3)		310 (17.7)
Ethnicity					
White	472 (89.7)		300 (92.6)		1,477 (84.4)
Afro-Caribbean	10 (1.9)		6 (1.9)		76 (4.3)
Asian	26 (4.9)		7 (2.2)		127 (7.3)
Mixed	5 (1.0)		0 (0.0)		25 (1.4)
Other	14 (2.7)		11 (3.4)		49 (2.8)
BMI (kg/m^3^)					
Mean (SD)	29.1 (6.5)	0.0001*	29.9 (6.0)	0.03	30.5 (6.4)
<18.5	6 (1.1)		2 (0.6)		5 (0.3)
18.5–24.9	127 (24.2)		63 (19.5)		280 (16.1)
25.0–29.9	193 (36.8)		121 (37.5)		649 (37.3)
≥30	201 (38.4)		138 (42.7)		819 (47.0)
Lung function					
FEV_1_ L (SD)	2.19 (0.68)		2.43 (0.64)		2.81 (0.74)
zFEV_1_ (SD)	−1.59 (0.99)	0.0001*	−0.69 (0.89)	0.0001*	−0.32 (0.98)
FVC L (SD)	3.73 (1.03)		3.60 (0.94)		3.62 (0.95)
zFVC (SD)	0.01 (1.11)	0.0001*	0.07 (1.04)	0.58	−0.26 (1.00)
FEV_1_/FVC (SD)	0.59 (0.08)		0.67 (0.02)		0.78 (0.05)
zFEV_1_/FVC	−2.48 (0.68)	0.0001*	−1.28 (0.26)	0.0001*	−0.16 (0.72)
Pack years					
Mean (SD)	25.2 (21.4)	0.0001*	25.3 (27.6)	0.0001*	16.9 (20.1)
Smoking status					
Never	71 (13.5)		53 (16.6)		422 (24.4)
Former	212 (40.5)		207 (64.9)		843 (48.6)
Current	241 (46.0)		59 (18.5)		468 (27.0)

**Notes:** z prefix denotes the *z*-score for the lung function index.

a Unless otherwise specified. The results for LLN−/FR+ were compared with LLN+/FR+ ($) and results for LLN+/FR+ were compared with LLN−/FR− (ψ) using Pearson Chi-square test for sex differences and Kruskal–Wallis tests for the other indices.

* *p*-values ≤0.001 are significant in accordance with Bonferroni correction for multiple comparisons.

**Abbreviations:** FEV_1_, forced expiratory volume in 1s; FR, fixed ratio; FVC, forced vital capacity; LLN, lower limit of normal; BMI, body mass index.

**Table 2:** Self-reported symptoms, comorbidities and quality of life, stratified by diagnostic criteria

	LLN+/FR+		LLN−/FR+		LLN−/FR−
	N (%)^a^	*p*-value^ψ^	N (%)^a^	*p*-value^$^	N (%)^a^
Subjects	527		324		1,753
Symptoms					
Wheeze	420 (80.5)	<0.001*	221 (69.3)	0.001*	1,133 (65.6)
Sputum	314 (60.9)	<0.001*	141 (44.8)	0.001*	843 (49.3)
Dyspnea	399 (75.7)	0.063	231 (71.3)	0.145	1,255 (71.6)
Cough	309 (59.7)	0.001*	138 (41.4)	0.001*	743 (43.1)
Chr cough	152 (28.8)	0.002	67 (20.7)	0.008	373 (21.3)
Chr sputum	118 (22.4)	0.001*	49 (15.6)	0.145	276 (15.7)
Comorbidities					
Heart disease	36 (6.9)	0.109	46 (14.2)	0.001*	159 (9.1)
Heart failure	10 (1.9)	0.086	13 (4.0)	0.068	59 (3.4)
Hypertension	205 (39.2)	0.697	161 (49.7)	0.003	699 (40.1)
Diabetes	59 (11.3)	0.022	58 (17.9)	0.007	266 (15.3)
Stroke	19 (3.6)	0.129	20 (6.2)	0.088	42 (2.4)
Depression	114 (21.8)	0.776	36 (11.1)	0.001*	390 (22.4)
Asthma	163 (30.9)	<0.001*	74 (22.8)	0.009	322 (18.4)
mMRC dyspnoea					
Median (IQR)	1 (1–2)	0.002	1 (0–2)	0.092	1 (0–2)
0	120 (24.0)		91 (29.1)		486 (28.9)
1	173 (34.5)		111 (35.5)		628 (37.3)
2	87 (17.4)		39 (12.5)		227 (13.5)
3	74 (14.8)		46 (14.7)		219 (13.0)
4	47 (9.4)		26 (8.3)		122 (7.3)
CAT score					
Median (IQR)	12 (7–19)	0.001*	10 (6–16)	0.001*	11 (6–16)
0–10	205 (43.9)		149 (53.6)		756 (49.5)
11–20	174 (37.3)		91 (32.7)		555 (36.4)
21–30	71 (15.2)		32 (11.5)		187 (12.3)
31–40	17 (3.6)		6 (2.2)		28 (1.8)
EQ-5D					
Median (IQR)	0.80 (0.68–1.0)	0.80	0.77 (0.64–0.91)	0.013	0.80 (0.68–1.0)

**Notes:**

a Unless otherwise specified. Results for LLN−/FR+ were compared with LLN+/FR+ ($) and results for LLN+/FR+ were compared with LLN−/FR− (ψ) using Pearson chi-square tests.

* *p*-values ≤0.001 are statistically significant in accordance with Bonferroni correction for multiple comparisons.

**Abbreviations:** CAT, COPD Assessment Test; Chr, chronic; EQ-5D, EuroQol-5D (measure of quality of life); FR, fixed ratio; IQR, interquartile range; LLN, lower limit of normal; mMRC, modified Medical Research Council score.

**Table 3:** Logistic regression model evaluating the association between risk of heart disease and presence of airflow limitation by different criteria

	Model 1 (n=2,589)	Model 2 (n=2,561)	Model 3 (n=2,559)

	OR (95% CI)	OR (95% CI)	OR (95% CI)
Sex			
Female	1.00	1.00	1.00
Male	1.98 (1.51–2.59)	2.03 (1.54–2.67)	1.92 (1.46–2.54)
Age	1.08 (1.06–1.10)	1.07 (1.06–1.09)	1.07 (1.05–1.09)
Diagnostic group			
LLN−/FR−	1.00	1.00	1.00
LLN−/FR+	0.85 (0.60–1.21)	0.87 (0.61–1.23)	0.90 (0.63–1.29)
LLN+/FR+	0.57 (0.40–0.81)	0.59 (0.41–0.85)	0.62 (0.43–0.90)
Smoking status			
Never		1.00	1.00
Former		0.99 (0.72–1.38)	0.98 (0.71–1.37)
Current		0.87 (0.57–1.32)	0.89 (0.58–1.36)
Co-morbidities			
Diabetesmellitus			1.71 (1.26–2.32)
Hypertension			1.73 (1.32–2.27)

**Notes:** Heart disease was defined by the presence of self-reported heart disease (n=241) or heart failure (n=82) or both (n=35). The LLN−/FR− group was the reference group with Model 1 adjusted for sex and age, Model 2 also adjusted for smoking status, and Model 3 also adjusted for diabetes mellitus and hypertension (n=2,559 with complete data).

**Abbreviations:** FR, fixed ratio (FEV_1_/FVC<0.7); LLN, lower limit of normal (FEV_1_/FVC<5th percentile); OR, odds ratio; FEV_1_, forced expiratory volume in 1 s; FVC, forced vital capacity.
